# Carbonylonium ions: the onium ions of the carbonyl group

**DOI:** 10.3762/bjoc.14.233

**Published:** 2018-10-04

**Authors:** Daniel Blanco-Ania, Floris P J T Rutjes

**Affiliations:** 1 Institute for Molecules and Materials, Radboud University, Heyendaalseweg 135, 6525 AJ Nijmegen, The Netherlands

**Keywords:** carboxonium ion, glycosylium ion, oxacarbenium ion, oxocarbenium ion, oxycarbenium ion

## Abstract

The nomenclature of cations R^1^C(=O^+^R^3^)R^2^ (R^1^, R^2^, R^3^ = H or organyl) has been examined and shown to be in a state of immeasurable confusion: a pragmatic recommendation is made that the generic term “carbonylonium ions” should be adopted for these intermediates, which comprises the terms “aldehydium” (R^1^ = H, R^2^, R^3^ = H or organyl) and “ketonium ions” (R^1^, R^2^ = organyl, R^3^ = H or organyl) for the corresponding aldehyde- and ketone-based intermediates, respectively.

## Introduction

**Figure 1:**
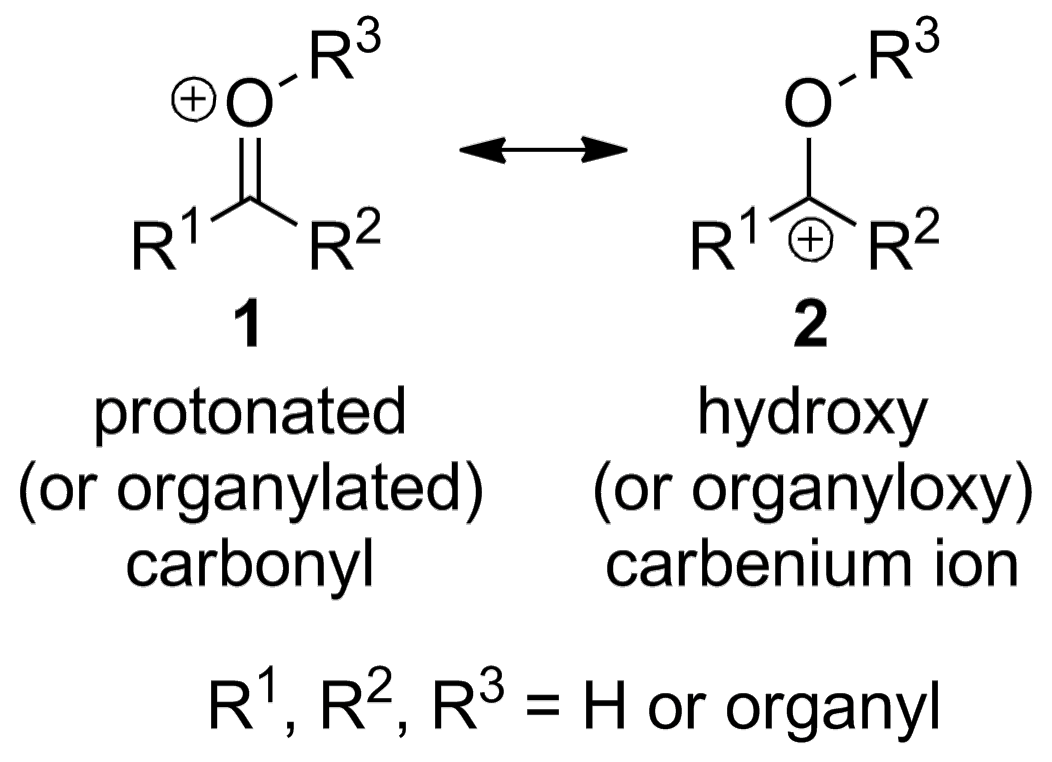
Protonated (or organylated) carbonyl (**1**) and hydroxy (or organyloxy) carbenium ion (**2**): two possible representations of the same intermediate.

There is much confusion in the literature over the name of the intermediates R^1^C(=O^+^R^3^)R^2^ (R^1^, R^2^, R^3^ = H or organyl [1], **1**; Figure 1).

In principle, these intermediates could be represented by the canonical form with a carbon–oxygen double bond, that is, a protonated (or organylated) carbonyl (**1**) or as a hydroxy (or organyloxy) carbenium ion (**2**; the term carbenium ion is used for R_3_C^+^: a structure, real or hypothetical, representing a carbocation which contains at least one carbon atom having only six valence electrons [2]; Figure 1). The best way to represent these intermediates according to the literature is as a protonated (or organylated) carbonyl (**1**) [3–4], therefore the names given in the literature have mostly tried to represent this resonance structure.

**Scheme 1:**
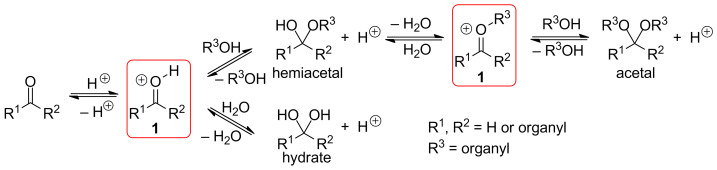
Acid-catalyzed interconversion of carbonyls, hydrates, hemiacetals and acetals.

These common intermediates are mainly found in acid-catalyzed reactions of aldehydes, ketones, hydrates, hemiacetals and acetals [5], that is, by protonation of a carbonyl group (aldehyde or ketone) or by detachment of a nucleofuge from the alpha position of an alcohol or an ether (Scheme 1).

One of the fields of Organic Chemistry more concerned on giving names to these intermediates has been carbohydrate chemistry. Its researchers have created different terms throughout history looking for a unique, concise and generic name for these kinds of intermediates found when glycosidation reactions take place [6]. “Oxacarbenium ions” [7–11], “oxocarbenium ions” [12–16], “oxycarbenium ions” [17–21], and “carboxonium ions” [22–23] are the most extended terms among the different names found in the literature for these intermediates in this and other fields of Organic Chemistry [24]. All these terms are ambiguous and none describes the actual structure of these intermediates. Some of these terms generated long ago when nomenclature was not as developed as it is today or were created without any sense of the etymology of the combined prefix and suffix. Needless to say that all the names that use the suffix “carbenium” do not represent the actual intermediates with all the atoms fulfilling the octet rule. Independently of the prefix used for this particular suffix, these names would refer to the canonical form with a trivalent carbon atom with six valence electrons or a modification thereof.

## Discussion

### Oxacarbenium ions

**Figure 2:**
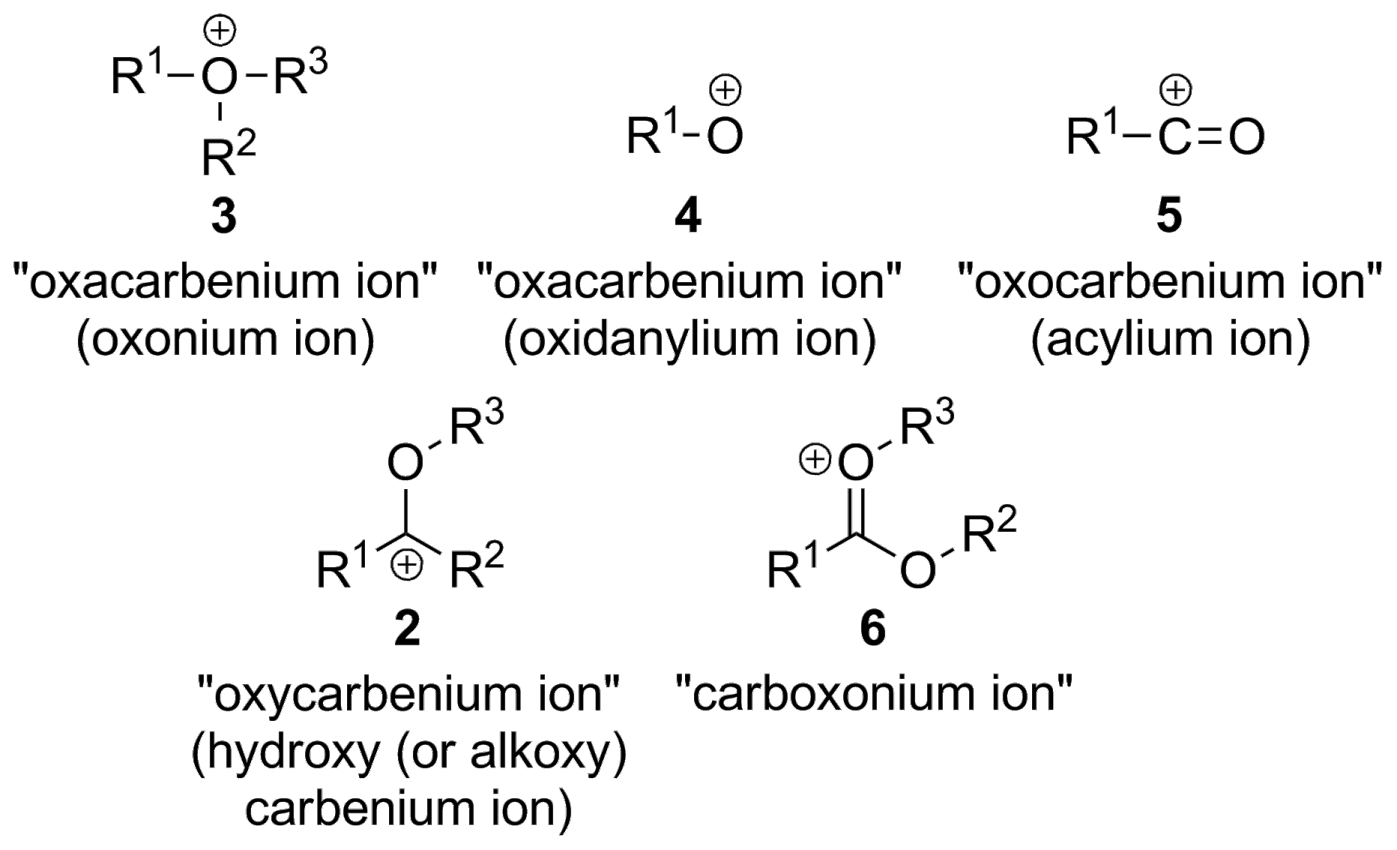
Oxacarbenium, oxocarbenium, oxycarbenium and carboxonium ions.

Firstly, “skeletal replacement” nomenclature (and also in Hantzsch–Widman nomenclature, but this kind of nomenclature is not applicable to the concerned intermediates) uses the “a” prefixes “oxa”, “aza”, “thia”… [25] for the replacement of carbon atoms by the heteroatoms oxygen, nitrogen and sulfur, respectively. Thus, “oxacarbenium ion” would denote a carbenium ion whose carbon atom is replaced by an oxygen atom, that is, an oxonium ion (**3**; Figure 2). Although if a coherent structure by formal subtraction of hydride from the parent structure was meant (note the use of carbenium and not carbonium ion), then an oxidanylium ion (**4**) would result (Figure 2). Either way, this term is not appropriate to describe intermediate **1**.

### Oxocarbenium ions

Secondly, the term “oxocarbenium ion” presents additional confusion because this term is also used to describe other intermediates. For example, the prefix “oxo” denotes the characteristic group “=O” [26–27] and, consequently, “oxocarbenium ion” is a carbenium ion with the group “=O” (i.e., a synonym of an acylium cation, **5**; Figure 2), which is the other use found in the literature for this term [23,28]. Not only is the term “oxocarbenium ion” not suitable for describing intermediate **1**, but also it is used to name another intermediate more accurately.

### Oxycarbenium ions

Thirdly, another term that presents confusion because it is used for different intermediates is “oxycarbenium ion”. This term is the closest term to describe correctly intermediates **1**, although it does not describe the canonical form with the positively-charged oxygen doubly-bonded to carbon, but the other resonance structure (**2**; Figure 1). The prefix “oxy” is used in nomenclature for the additive operation called “concatenation” where “oxy” represents the group “–O–” [29], e.g., acyloxy (RCO–O–), alkyloxy (R–O–) and hydroxy (H–O–). Sometimes the prefix “oxy” is used to represent all the “oxy”-ending groups, like in “oxymercuriation” for the addition of an acyloxy, alkyloxy or hydroxy group alongside a group with mercury to a double bond. “Oxycarbenium ion” can also be found in the literature with this meaning that it correctly describes the same intermediate, although not the more realistic representation of it [30].

### Carboxonium ions

Other research groups, e.g., Olah’s and Prakash’s, have used the term “carboxonium ion” to describe intermediates **1**. The issue with this term is that it is used for many different oxonium ions independently of their structure, mainly intermediates with a variable number of oxygen atoms bound to the central carbon atom. This name is used to describe protonated carboxylic acids (carboxylic acidium ions), protonated esters and protonated aldehydes and ketones amongst others [31]. The name thought to be originated from the combination of the terms “carbenium” and “oxonium”, but not correctly applied to such a broad scope of intermediates. Clearly the intermediates formed from protonation of carboxylic acids and esters are different than the ones from aldehydes and ketones and therefore should not have the same name. We recommend applying “carboxonium ion” only to intermediates whose carbon atom presents the same oxidation state as carboxylic acids, that is, protonated carboxylic acids and esters (**6**; Figure 2), in agreement with other terms like “carboxy”, “carboxylic”, “carboxylate” and “carboxamide”. To augment confusion, other researchers have used this term to describe “oxycarbenium ions” **2** [32–33].

### Carbonylonium ions: aldehydium and ketonium ions

We proceed then to propose a precise and usable term for describing intermediates **1** after having demonstrated that the names used in the literature are inaccurate. Modern nomenclature is able to name and describe every single canonical form of any compound independently of the complexity of the intermediate. Applying systematic nomenclature to intermediates **1** would generate the cumbersome names “organylidene oxonium ions” or “organylidene oxidanium ions” [34]. Clearly, the length and complexity of these names would prevent researchers from using them. Another alternative would be to combine the terms “carbene” (divalent carbon atom bound to a parent hydride) and “oxonium ion” (or “oxidanium ion”) forming “carbenoxonium ions” (or “carbenoxidanium ions”). These other terms are as long as the names currently used in the literature commented above, but they lack the stem of the functional group they come from and from our point of view researchers would not grasp the structure of **1** at a glance. Therefore, the need of coining a new, easy and accurate term is crying out.

Simple names of protonated functional groups (or the corresponding organyl derivatives) based on the stem of the name of the functional group followed by a suffix indicating the positively-charged groups has made nomenclature richer and more fluent. IUPAC has given name to several functional groups as protonated species and organyl derivatives thereof: imine–iminium, amide–amidium, nitrile–nitrilium, amine–aminium [34]. In this sense, we propose the use of “aldehydium” and “ketonium ions” (forlorn names by IUPAC probably because they cannot be used in systematic nomenclature) for protonated aldehydes and ketones, respectively, terms shyly used in the literature [35–37]. Finally, the only remaining matter would be to give a name to intermediates **1** as a whole: a generic term for a protonated (or organylated) carbonyl. A straightforward name would be derived from “carbonyl” plus one of the two suffixes used for naming positively-charged molecular entities: “ium” or “onium”. Because “carbonylium ion” is already used for naming some of intermediates **5** [38], we therefore propose the new term “carbonylonium ion”. We reckon that this term unambiguously describes the structure of intermediates **1**, researchers would immediately visualize its structure with a glimpse and there would not be any confusion because of its novelty it has not been used for any other intermediate. This term is also consistent with names such as “diazonium” and “uronium ions”.

## Conclusion

We have coined the new term “carbonylonium ions” to name the intermediates R^1^C(=O^+^R^3^)R^2^ (R^1^, R^2^, R^3^ = H or organyl). We believe this unequivocal name could end the historic confusion carried by the nomenclature of these intermediates. We hope that this name is integrated soon in the vocabulary of Organic Chemistry. We also propose the use of the terms “aldehydium” and “ketonium ions” to specify the nature (aldehyde or ketone, respectively) of these intermediates.
